# Association of combined waist-to-height ratio and systolic blood pressure categories with incident cardiovascular disease in middle-aged and older adults

**DOI:** 10.1038/s41598-026-55572-z

**Published:** 2026-05-28

**Authors:** Jing Wang, Xu Liu

**Affiliations:** 1 Department of Social Services, Shengjing Hospital Affiliated to China Medical University, Shenyang, Liaoning China; 2 Department of Cardiology, The Second Affiliated Hospital of Shenyang Medical College, No 20 Beijiu Road, Heping District, Shenyang, Liaoning China

**Keywords:** Waist-to-height ratio, Systolic blood pressure, Cardiovascular risk stratification, Community-based screening, CHARLS, Cardiology, Diseases, Health care, Medical research, Risk factors

## Abstract

Cardiovascular disease (CVD) is the leading cause of global mortality. Early identification of individuals at higher risk of CVD is important for prevention and management. Traditional cardiovascular risk assessment approaches often require laboratory testing and related infrastructure, which may limit their implementation in community settings. This prospective cohort study examined the association of combined waist-to-height ratio (WHtR) and systolic blood pressure (SBP) categories with incident CVD in middle-aged and older adults. We analyzed 6,770 adults aged ≥ 45 years without baseline CVD from the China Health and Retirement Longitudinal Study (CHARLS, 2011–2018). Participants were categorized by WHtR (≥ 0.5/<0.5) and SBP (≥ 140/<140 mmHg). Cox proportional hazards models were used to assess associations with incident CVD, adjusting for sociodemographic, lifestyle, and metabolic covariates. Over 7 years of follow-up, 1,384 CVD events occurred. Elevated WHtR alone (HR = 1.271, 95% CI: 1.111–1.455) and elevated SBP alone (HR = 1.392, 95% CI: 1.240–1.564) were each associated with a higher risk of incident CVD. Participants with concurrent elevations in WHtR and SBP had a 68.2% higher risk of incident CVD (HR = 1.682, 95% CI: 1.417–1.996) compared with those with normal values. These associations remained consistent across subgroup and sensitivity analyses. Combined WHtR and SBP categories may offer a simple, low-cost approach for community-based cardiovascular risk stratification in middle-aged and older adults. Requiring only a tape measure and a sphygmomanometer, this approach may help identify individuals at higher risk of incident CVD in settings where laboratory testing is less accessible.

**Supplementary Information:**

The online version contains supplementary material available at 10.1038/s41598-026-55572-z.

## Introduction

Cardiovascular disease (CVD) remains the leading cause of global mortality and disability, with Asia bearing a disproportionate burden driven by rapid population aging and the rise in metabolic disorders. By 2050, CVD prevalence in Asia is projected to exceed 729.5 million cases—a 109% increase from 2025—while annual cardiovascular deaths may surpass 24.1 million^1^. These alarming figures underscore the urgent need for effective early-risk detection tools, particularly in community settings where timely intervention can significantly reduce morbidity and mortality. Established models, such as the Framingham Risk Score^2^ and the ASCVD Risk Calculator^3^, offer robust assessments but remain largely impractical in community care settings due to their reliance on laboratory inputs. This highlights the need for noninvasive, simple, and easily accessible tools that can be readily implemented in community-based primary care.

Recent studies have highlighted growing interest in integrated cardiometabolic markers that combine routinely available measures to improve cardiovascular risk stratification. For example, the uric acid-to-high-density lipoprotein cholesterol ratio (UHR) has been associated with long-term cardiovascular risk and mortality, whereas the non-high-density lipoprotein cholesterol-to-high-density lipoprotein cholesterol ratio (NHHR) has also shown significant associations with cardiovascular conditions such as angina pectoris^4,5^. Nevertheless, these ratio-based indices still depend on laboratory-derived lipid or biochemical measurements, which may limit their scalability in resource-constrained community settings. By contrast, a fully non-laboratory combination integrating an anthropometric index with a bedside hemodynamic measure may offer a more accessible and scalable option for community-based cardiovascular risk stratification.

The waist-to-height ratio (WHtR) and systolic blood pressure (SBP) are two well-established, field-applicable indicators for cardiovascular risk assessment. WHtR, calculated as waist circumference divided by height, has demonstrated superior performance over body mass index (BMI) and waist circumference in detecting central obesity and identifying cardiometabolic risk^6^. A universal cutoff of WHtR ≥ 0.5 is strongly associated with elevated risks of hypertension, diabetes, and dyslipidemia, establishing its practicality for large-scale screening^7^. Although sex-specific thresholds (≥ 0.51 for men and ≥ 0.53 for women) have been proposed^8^, the pragmatic cutoff of 0.5 remains recommended in Chinese clinical guidelines^9^.

Likewise, elevated SBP independently amplifies cardiovascular risk: each 10-mmHg increase is associated with a 45% higher risk of ischemic heart disease and a 65% higher risk of stroke among middle-aged adults^10^. In China, elevated SBP is estimated to contribute to 2.67 million annual cardiovascular deaths^11^, emphasizing an urgent need for effective prevention strategies. Although both WHtR and SBP have been individually recognized as important indicators of cardiovascular risk, prospective evidence on their combined use for community-based risk stratification remains limited.

To address this gap, our study used data from the China Health and Retirement Longitudinal Study (CHARLS) to examine the association of combined WHtR and SBP categories with incident CVD. This approach requires only basic tools—a tape measure and a sphygmomanometer—and may therefore represent a practical, noninvasive strategy for identifying higher-risk individuals in community settings, particularly where access to laboratory testing is limited.

## Results

### Baseline characteristics

**Fig. 1:**
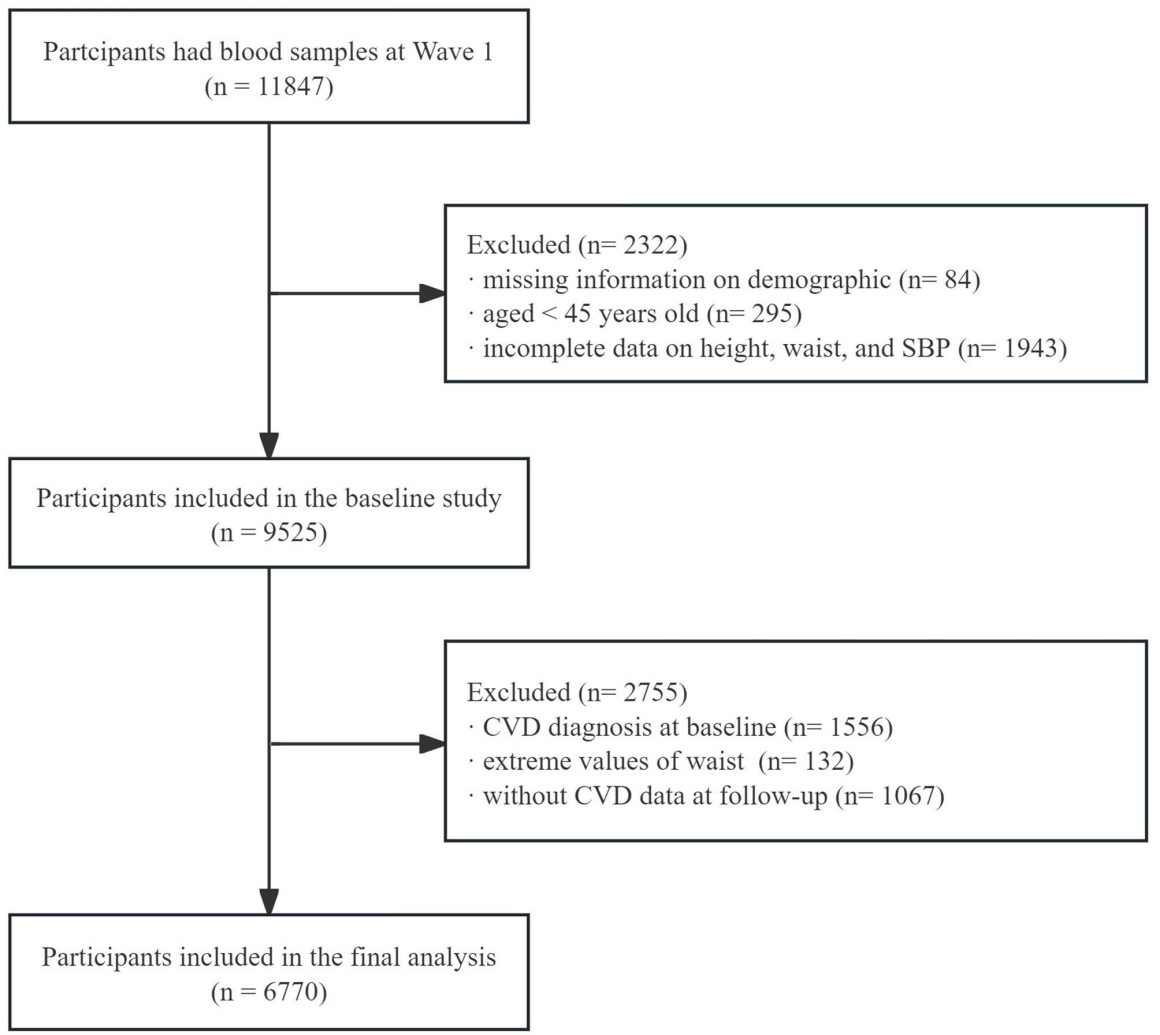
Flow diagram for participants included in the study.

This study included 6,770 participants (53.6% female, mean age 58.24 ± 8.86 years), categorized into four groups based on WHtR (< 0.5 or ≥ 0.5) and SBP (< 140 mmHg or ≥ 140 mmHg). As shown in Table 1, there were significant differences across groups for most baseline characteristics (all *P* < 0.001), except for kidney and liver diseases. Group 4 (WHtR ≥ 0.5 and SBP ≥ 140 mmHg) exhibited the highest cardiovascular risk, characterized by the largest WHtR, highest blood pressure levels, and highest prevalence of metabolic disorders (hypertension 50.9%, diabetes 8.0%, dyslipidemia 11.8%). In contrast, Group 1 (WHtR < 0.5 and SBP < 140 mmHg) demonstrated the most favorable metabolic and cardiovascular profiles. Furthermore, Table S2 summarizes the baseline characteristics of participants grouped by CVD status. Participants in the CVD group were older and exhibited significantly poorer metabolic profiles compared to those without CVD (all *P* < 0.05) (Fig. 1).

**Table 1:** Baseline characteristics of participants.

Characteristics	Overall	Group 1	Group 2	Group 3	Group 4	*P* value
N	6770	1666	3379	330	1395	
Gender, n (%)						< 0.001
Male	3139 (46.4)	1075 (64.5)	1286 (38.1)	216 (65.5)	562 (40.3)	
Female	3631 (53.6)	591 (35.5)	2093 (61.9)	114 (34.5)	833 (59.7)	
Age, years	58.24 ± 8.86	57.41 ± 8.47	57.02 ± 8.28	62.12 ± 9.74	61.29 ± 9.48	< 0.001
Marital status, n (%)						< 0.001
Married	5759 (85.1)	1453 (87.2)	2910 (86.1)	250 (75.8)	1146 (82.2)	
Other	1011 (14.9)	213 (12.8)	469 (13.9)	80 (24.2)	249 (17.8)	
Education level, n (%)						< 0.001
Elementary school or below	4757 (70.3)	1119 (67.2)	2317 (68.6)	257 (77.9)	1064 (76.3)	
Middle school	1841 (27.2)	509 (30.6)	969 (28.7)	65 (19.7)	298 (21.4)	
College or above	172 (2.5)	38 (2.3)	93 (2.8)	8 (2.4)	33 (2.4)	
Residence, n (%)						< 0.001
Rural	5773 (85.3)	1484 (89.1)	2804 (83.0)	298 (90.3)	1187 (85.1)	
Urban	997 (14.7)	182 (10.9)	575 (17.0)	32 (9.7)	208 (14.9)	
Smoking status, n (%)						< 0.001
Never	4126 (60.9)	781 (46.9)	2310 (68.4)	136 (41.2)	899 (64.4)	
Former	522 (7.7)	126 (7.6)	269 (8.0)	25 (7.6)	102 (7.3)	
Current	2122 (31.3)	759 (45.6)	800 (23.7)	169 (51.2)	394 (28.2)	
Drinking status, n (%)						< 0.001
Never	3956 (58.4)	814 (48.9)	2127 (62.9)	158 (47.9)	857 (61.4)	
Former	524 (7.7)	124 (7.4)	239 (7.1)	27 (8.2)	134 (9.6)	
Current	2290 (33.8)	728 (43.7)	1013 (30.0)	145 (43.9)	404 (29.0)	
Hypertension, n (%)	1486 (21.9)	107 (6.4)	554 (16.4)	115 (34.8)	710 (50.9)	< 0.001
Diabetes, n (%)	333 (4.9)	34 (2.0)	182 (5.4)	6 (1.8)	111 (8.0)	< 0.001
Dyslipidemia, n (%)	494 (7.3)	53 (3.2)	267 (7.9)	10 (3.0)	164 (11.8)	< 0.001
Kidney disease, n (%)	326 (4.8)	91 (5.5)	153 (4.5)	19 (5.8)	63 (4.5)	0.385
Liver disease, n (%)	194 (2.9)	52 (3.1)	103 (3.0)	12 (3.6)	27 (1.9)	0.120
Antihypertensive treatment, n (%)	1037 (15.3)	69 (4.1)	400 (11.8)	64 (19.4)	504 (36.1)	< 0.001
Antihyperglycemic treatment, n (%)	202 (3.0)	18 (1.1)	118 (3.5)	4 (1.2)	62 (4.4)	< 0.001
Lipid-lowering treatment, n (%)	232 (3.4)	18 (1.1)	123 (3.6)	6 (1.8)	85 (6.1)	< 0.001
SBP, mmHg	128.53 ± 20.90	116.52 ± 12.31	120.01 ± 11.68	156.31 ± 14.29	156.92 ± 14.96	< 0.001
DBP, mmHg	74.97 ± 11.98	69.29 ± 9.67	71.95 ± 9.37	85.85 ± 11.47	86.51 ± 10.95	< 0.001
BMI, kg/m^2^	23.08 (20.84, 25.62)	20.23 (18.98, 21.58)	24.19 (22.38, 26.32)	20.17 (18.75, 21.60)	24.97 (22.78, 27.39)	< 0.001
FBG, mg/dl	102.24 (94.32, 112.86)	99.18 (91.98, 108.50)	102.60 (94.68, 113.58)	102.06 (94.95, 112.14)	105.12 (96.30, 117.99)	< 0.001
HbA1c, %	5.10 (4.90, 5.40)	5.10 (4.80, 5.30)	5.20 (4.90, 5.50)	5.10 (4.80, 5.40)	5.20 (4.90, 5.50)	< 0.001
TG, mg/dl	104.43 (74.34, 152.22)	82.31 (62.83, 119.47)	110.62 (79.65, 161.96)	92.04 (65.49, 131.64)	122.13 (85.85, 179.21)	< 0.001
TC, mg/dl	190.59 (167.40, 215.34)	182.86 (161.21, 206.44)	192.14 (168.56, 216.88)	190.01 (167.11, 213.98)	196.39 (171.65, 222.29)	< 0.001
HDL-c, mg/dl	51.47 ± 15.36	56.38 ± 15.84	49.64 ± 14.52	57.38 ± 16.65	48.58 ± 14.65	< 0.001
LDL-c, mg/dl	116.17 ± 34.86	111.17 ± 32.20	117.63 ± 35.06	112.43 ± 32.31	119.91 ± 37.20	< 0.001
WHtR	0.53 (0.49, 0.58)	0.47 (0.45, 0.49)	0.56 (0.53, 0.59)	0.47 (0.45, 0.49)	0.58 (0.54, 0.62)	< 0.001

SBP, systolic blood pressure; DBP, diastolic blood pressure; BMI, body mass index; FBG, fasting blood glucose; HbA1c, glycosylated hemoglobin A1c; TG, triglycerides; TC, total cholesterol; HDL-c, high-density lipoprotein cholesterol; LDL-C, low-density lipoprotein cholesterol; WHtR, waist-to-height ratio.

### Association of WHtR and SBP with CVD incidence risk

**Fig. 2:**
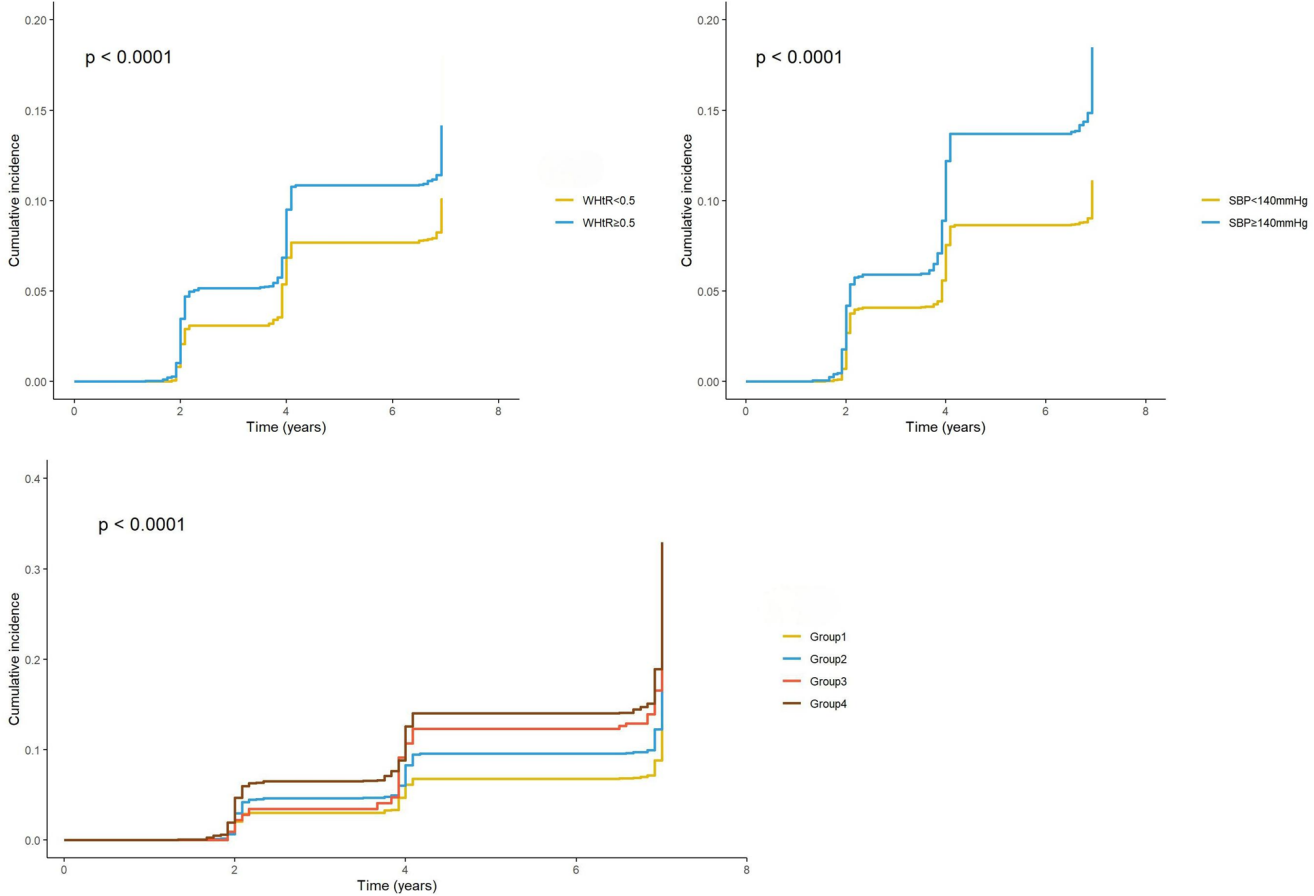
Kaplan-Meier plot of CVD by WHtR and SBP thresholds. CVD, cardiovascular disease; WHtR, waist-to-height ratio; SBP, systolic blood pressure.

Over a 7-year follow-up period, 1,384 CVD events were recorded, accounting for 20.4% of participants. Kaplan-Meier survival analysis revealed significantly higher cumulative CVD incidence among those with WHtR ≥ 0.5 and SBP ≥ 140 mmHg. A progressive increase in CVD incidence was observed across WHtR and SBP categories (Group 4 > Group 3 > Group 2 > Group 1, *P* < 0.001; Fig. 2), a trend consistent for heart conditions and strokes (Figures S1, S2). Multivariate Cox regression analysis showed that the associations remained directionally consistent across progressively adjusted models. In Model 3, after further adjustment for baseline metabolic covariates, participants with WHtR ≥ 0.5 had 1.271 times higher CVD risk (95% CI: 1.111–1.455) compared to those with WHtR < 0.5, and participants with SBP ≥ 140 mmHg had 1.392 times higher CVD risk (95% CI: 1.240–1.564). Compared to Group 1, participants in Group 4 had the highest CVD risk (HR = 1.682, 95% CI: 1.417–1.996; Table 2). Further analyses confirmed that elevated WHtR, elevated SBP, and their combination were consistently associated with increased risks of heart conditions and strokes. After further adjustment for baseline metabolic covariates in Model 3, Group 4 participants had significantly higher risks of heart conditions (HR = 1.444, 95% CI: 1.185–1.759) and strokes (HR = 3.041, 95% CI: 2.236–4.137), compared to the reference group (Tables S3, S4).

**Table 2:** Association of WHtR and SBP with CVD incidence risk.

	Case, *n* (%)	Crude		Model 1		Model 2		Model 3	
		HR (95% CI)	*P* value	HR (95% CI)	*P* value	HR (95% CI)	*P* value	HR (95% CI)	*P* value
**WHtR**									
WHtR < 0.5	312 (15.6)	Reference		Reference		Reference		Reference	
WHtR ≥ 0.5	1072 (22.5)	1.494 (1.317, 1.694)	< 0.001	1.428 (1.255–1.626)	< 0.001	1.378 (1.209–1.570)	< 0.001	1.271 (1.111–1.455)	< 0.001
**SBP**									
SBP < 140 mmHg	917 (18.2)	Reference		Reference		Reference		Reference	
SBP ≥ 140 mmHg	467 (27.1)	1.652 (1.478–1.847)	< 0.001	1.460 (1.302–1.639)	< 0.001	1.471 (1.311–1.651)	< 0.001	1.392 (1.240–1.564)	< 0.001
**WHtR and SBP**									
Group 1	238 (14.3)	Reference		Reference		Reference		Reference	
Group 2	679 (20.1)	1.443 (1.245–1.673)	< 0.001	1.398 (1.202–1.626)	< 0.001	1.348 (1.158–1.569)	< 0.001	1.277 (1.094–1.491)	0.002
Group 3	74 (22.4)	1.810 (1.394–2.351)	< 0.001	1.595 (1.222–2.081)	< 0.001	1.623 (1.241–2.122)	< 0.001	1.591 (1.222–2.071)	< 0.001
Group 4	393 (28.2)	2.222 (1.891, 2.610)	< 0.001	1.926 (1.627–2.280)	< 0.001	1.871 (1.580–2.216)	< 0.001	1.682 (1.417–1.996)	< 0.001

WHtR, waist-to-height ratio; SBP, systolic blood pressure; CVD, cardiovascular disease. HR, hazard ratio; CI, confidence interval. Crude: unadjusted model; Model 1: adjusted for age, gender; Model 2: adjusted for marital status, education level, residence, smoking status and drinking status based on Model 1; Model 3: additionally adjusted for HbA1c, HDL-c, LDL-c, diabetes, and dyslipidemia to account for baseline metabolic status, based on Model 2.

### Interaction analysis between WHtR and SBP

After further adjustment for baseline metabolic covariates in Model 3, no significant multiplicative interaction between WHtR and SBP was observed in relation to incident CVD (HR for interaction = 0.828, 95% CI: 0.619–1.106; *P* = 0.202). Additive interaction analyses were also not significant (RERI = − 0.186, 95% CI: −0.626 − 0.254; AP = − 0.111, 95% CI: −0.373 − 0.151; S = 0.786, 95% CI: 0.297–2.076).

### Subgroup analysis

We conducted a stratified analysis to evaluate the association between the combined WHtR and SBP-based groupings and the risk of CVD incidence across various subgroups. As shown in Table 3, none of the stratified subgroups, including age, gender, current smoking status, current drinking status, as well as the presence of hypertension, diabetes, and dyslipidemia, significantly altered the relationship between WHtR and SBP-based groupings and CVD incidence (all P for interaction > 0.05). Notably, in the subgroup of participants with diabetes, the wide confidence interval for Group 3 (HR = 3.757, 95% CI: 0.940-15.023) suggests limited precision due to the small sample size, which warrants cautious interpretation.

**Table 3:** Subgroup analysis for the association of the WHtR and SBP on CVD incidence.

	Group 1	Group 2	Group 3	Group 4	*P* for interaction
Age					0.128
< 60	Ref	1.549 (1.262–1.902)	1.670 (1.093–2.552)	2.476 (1.956–3.135)	
≥ 60	Ref	1.346 (1.088–1.665)	1.550 (1.108–2.168)	1.715 (1.373–2.141)	
Gender					0.857
Male	Ref	1.414 (1.150–1.739)	1.886 (1.363–2.607)	2.245 (1.786–2.822)	
Female	Ref	1.343 (1.075–1.678)	1.572 (1.011–2.443)	1.992 (1.570–2.528)	
Smoking					0.627
Yes	Ref	1.277 (0.990–1.648)	1.795 (1.233–2.612)	2.241 (1.710–2.937)	
No	Ref	1.452 (1.202–1.755)	1.744 (1.212–2.511)	2.122 (1.728–2.605)	
Drinking					0.348
Yes	Ref	1.515 (1.179–1.949)	1.454 (0.933–2.264)	2.100 (1.577–2.798)	
No	Ref	1.346 (1.120–1.618)	2.007 (1.453–2.772)	2.109 (1.731–2.569)	
Hypertension					0.953
Yes	Ref	1.281 (0.854–1.920)	1.311 (0.788–2.180)	1.518 (1.021–2.258)	
No	Ref	1.339 (1.140–1573)	1.530 (1.095–2.137)	1.653 (1.335–2.047)	
Diabetes	Ref				0.645
Yes	Ref	1.860 (0.801–4.320)	3.757 (0.940−15.023 )	2.397 (1.018–5.646)	
No	Ref	1.401 (1.205–1.629)	1.724 (1.322–2.249)	2.135 (1.809–2.520)	
Dyslipidemia	Ref				0.639
Yes	Ref	1.436 (0.820–2.513)	2.592 (0.853–7.875)	1.818 (1.027–3.219)	
No	Ref	1.369 (1.174–1.598)	1.750 (1.338–2.288)	2.069 (1.744–2.455	

WHtR, waist-to-height ratio; SBP, systolic blood pressure; CVD, cardiovascular disease. Stratified analyses were performed based on Model 3.

### Sensitivity analysis

To assess the robustness of the primary findings, we conducted multiple sensitivity analyses, which consistently demonstrated similar results. First, excluding participants with missing data, the association between the combined WHtR and SBP-based groupings and CVD incidence remained consistent with the main findings (Table S4). Second, after excluding participants with a follow-up period of less than 2 years, the association remained unchanged (Table S5). Even after removing participants who were receiving antihypertensive or lipid-lowering treatments, the results persisted (Table S6).

## Discussion

In this prospective cohort of 6,770 Chinese participants aged 45 years and older, a 7-year follow-up revealed that concurrent elevations in WHtR (≥ 0.5) and SBP (≥ 140 mmHg) were significantly associated with a higher risk of incident CVD. After adjustment for sociodemographic and lifestyle factors, and after further accounting for baseline metabolic covariates, the combined risk profile was associated with a 68.2% higher risk of incident CVD (HR = 1.682, 95% CI: 1.417–1.996), exceeding the associations observed for either marker alone. These findings remained robust across subgroups and sensitivity analyses. Importantly, this noninvasive protocol—assessing WHtR and SBP—may represent a practical and cost-effective alternative to traditional risk assessment approaches that rely on laboratory testing. Based on findings from China’s nationally representative CHARLS cohort, our results suggest that this approach may offer a scalable option for community-based CVD risk stratification and may support primary care priorities under the “Healthy China 2030” initiative.

Despite the wide availability of cardiovascular risk assessment tools, their routine implementation in primary care remains challenging in settings where laboratory access is limited. Traditional multivariable equations—such as the Framingham Risk Score and the ASCVD algorithm^2,3^—as well as newer integrated markers (e.g., UHR and NHHR)^4,5^ continue to require biochemical testing. Against this background, the combined measurement of WHtR and SBP may represent a pragmatic, non-laboratory complementary approach for community-based risk stratification. Both indicators can be obtained rapidly using only a tape measure and a sphygmomanometer, and may help identify individuals who would benefit from closer cardiovascular evaluation in primary care practice.

The selection of WHtR as a core component in our study is grounded in its well-established role as a useful anthropometric indicator. WHtR provides a robust proxy for visceral adiposity^12^, and has been shown to outperform both BMI and waist circumference in identifying obesity-related cardiometabolic risk^6,13^. Meta-analyses have supported its utility across populations^6^, with a linear dose-response relationship reported between WHtR and CVD risk, such that each 5% increase in WHtR is associated with a 23% higher risk of ischemic CVD^14^. Studies in Asian cohorts have further supported the association of WHtR with long-term CVD risk^15^ and its potential utility in heart failure risk stratification^16^. In the present study, elevated WHtR was independently associated with incident CVD in middle-aged and older Chinese adults. This association may be explained, at least in part, by the ability of WHtR to capture visceral fat accumulation, a key contributor to metabolic imbalance and vascular dysfunction^17,18^. Visceral adiposity promotes a pro-inflammatory state through the secretion of cytokines such as interleukin-6 (IL-6) and tumor necrosis factor-α (TNF-α), contributing to systemic inflammation, insulin resistance, and endothelial dysfunction, all of which are well-established mechanisms in atherosclerosis and CVD^19^. Taken together, these findings support WHtR as a practical marker of cardiometabolic risk and early vascular impairment.

Complementing WHtR, SBP represents a major hemodynamic determinant of CVD. Prospective cohort studies across 61 populations have consistently demonstrated a dose-dependent relationship between SBP and CVD risk, with each 20-mmHg increase in SBP associated with an approximate doubling of CVD event risk^20^. In addition, SBP levels > 140 mmHg have been associated with a 50.8% higher cardiovascular risk^21^. Mendelian randomization studies further confirm this causal relationship, showing that genetically proxied 10-mmHg SBP elevations increase CVD risk by 49%^22^. Our study extends this evidence to middle-aged and older Chinese populations, showing that SBP ≥ 140 mmHg was associated with a 39.2% increase in CVD risk after further adjustment for baseline metabolic covariates. The pathophysiological interplay underlying this association involves hemodynamic stress (endothelial damage, arterial remodeling, accelerated atherosclerosis)^23,24^, and non-hemodynamic effects (vascular inflammation, oxidative stress, maladaptive neurohormonal activation)^25,26^. These processes collectively create a pro-inflammatory and pro-thrombotic state, further amplifying cardiovascular risk. Together, these processes may contribute to structural and functional changes in the cardiovascular system and thereby increase the likelihood of adverse cardiovascular outcomes.

Although both SBP and WHtR are established indicators of CVD risk, each captures only part of the multifactorial processes underlying cardiovascular risk. While elevated SBP primarily reflects hemodynamic stress, including arterial stiffness and endothelial injury^24^, it also may contribute to inflammatory processes^26^. Conversely, WHtR predominantly reflects visceral adiposity-driven pathways, including systemic inflammation and insulin resistance^19^. These distinct yet overlapping mechanisms suggest close links between metabolic and hemodynamic dysfunction. However, reliance on either metric alone may overlook important interplay between these domains. For instance, WHtR may fail to identify vascular risks in individuals with normal adiposity, while SBP alone cannot detect subclinical metabolic dysfunction. Taken together, these considerations support the rationale for integrated cardiovascular risk stratification, as the metabolic and hemodynamic information captured by WHtR and SBP may be complementary rather than interchangeable.

In the present study, the combined use of WHtR ≥ 0.5 and SBP ≥ 140 mmHg was associated with a higher risk of incident CVD than either metric alone. Compared to using either metric alone, concurrent elevations in these two indicators were associated with a 68.2% higher risk of incident CVD. Although participants with concurrent elevations in WHtR and SBP had the highest risk of incident CVD, formal interaction analyses did not support a statistically significant multiplicative or additive interaction between the two indicators. Although previous studies have separately supported the associations of visceral obesity (as represented by WHtR) and hypertension (as represented by SBP) with CVD risk, evidence regarding their combined use for community-based risk stratification remains limited, particularly among middle-aged and older populations. Our findings suggest that integrating these two noninvasive indicators may provide a practical approach to cardiovascular risk stratification in community care settings. Notably, the universally recommended WHtR cutoff value (≥ 0.5) showed consistent associations in this study, aligning with the standards of the NICE ( National Institute for Health and Care Excellence) obesity management guidelines^12^ and suggesting potential broader applicability across populations. The observed joint association of WHtR and SBP may reflect the coexistence of adverse metabolic and hemodynamic abnormalities. Specifically, visceral fat-derived pro-inflammatory cytokines (e.g., TNF-α, IL-6) and adipokines (e.g., leptin) drive systemic inflammation, contributing to insulin resistance and vascular damage^19^, while hypertension-induced oxidative stress and vascular remodeling promote arterial stiffness and lipid deposition^24,27^. These coexisting mechanisms may form a self-reinforcing cycle in which vascular injury accelerates atherosclerosis while worsening metabolic dysfunction. Accordingly, simultaneous attention to visceral adiposity and blood pressure may represent a pragmatic strategy for cardiovascular risk reduction.

Despite its strengths, this study has several limitations. First, the findings are based on Chinese adults aged 45 years and older, which may limit the generalizability to younger populations or other ethnic groups. Second, while SBP was measured in triplicate to enhance accuracy, WHtR relied on a single measurement of waist circumference and height, potentially introducing random error and attenuating risk estimates due to regression dilution bias. Third, CVD ascertainment relied on self-reported data, which may under-detect asymptomatic events such as silent myocardial ischemia. Fourth, the observational design precludes causal inferences, and residual confounding from unmeasured factors (e.g., diet, physical activity, or genetic predisposition) cannot be fully excluded. In addition, some metabolic covariates included in Model 3 may partly overlap with cardiometabolic pathways linking WHtR and SBP to CVD, which may have resulted in conservative effect estimates. Finally, the analysis did not incorporate emerging cardiovascular biomarkers or imaging parameters, which may further improve risk stratification in future studies.

## Conclusion

The combined use of WHtR (≥ 0.5) and SBP (≥ 140 mmHg) thresholds may represent a practical, low-cost approach for cardiovascular risk stratification in middle-aged and older Chinese adults, especially in community-based primary care settings. By capturing complementary hemodynamic and metabolic-inflammatory information, this approach was associated with a 68.2% higher risk of incident CVD without requiring laboratory testing. Using only a tape measure and a sphygmomanometer, it may help identify individuals at higher cardiovascular risk and may support community-based prevention strategies aligned with the Healthy China 2030 initiative. Future research should further assess its generalizability, implementation feasibility, and potential role in primary care risk stratification.

## Methods

### Study participants and design

This study utilized nationally representative prospective cohort data from the CHARLS, employing its multi-stage, stratified probability sampling design to recruit adults aged 45 years and older in China^28^. Between June 2011 and March 2012, the baseline survey recruited 17,708 participants from 450 communities/villages across 150 counties/districts in 28 provinces, forming a nationally representative sample. Data collection involved standardized face-to-face interviews conducted by trained health workers, using structured questionnaires. Follow-up surveys, conducted at two-year intervals, established a longitudinal framework for tracking participants over time. The study was approved by the Biomedical Ethics Review Committee of Peking University (IRB00001052-11015), and all participants provided written informed consent. This study followed the Strengthening the Reporting of Observational Studies in Epidemiology (STROBE) guidelines^29^. We confirm that all methods were carried out in accordance with relevant ethical guidelines.

For this study, 11,847 participants with available blood samples at baseline were initially included. Subsequently, 5,077 participants were excluded based on the following criteria: missing demographic data (*n* = 84), including age and gender; age < 45 years (*n* = 295); incomplete data on height, waist circumference, or systolic blood pressure (*n* = 1,943); diagnosed CVD at baseline (*n* = 1,556); extreme waist circumference values (< 40 cm or > 200 cm) (*n* = 132), as such measurements are biologically implausible and likely represent data entry errors; no CVD data during follow-up (*n* = 1,067). Consequently, 6,770 participants met the inclusion criteria and were considered for the final analysis. Figure 1 presents a detailed flowchart illustrating the inclusion and exclusion process.

### WHtR and blood pressure measurement

In this study, WHtR was calculated as the ratio of waist circumference to height. Trained investigators measured waist circumference using a soft measuring tape at the level of the umbilicus, while height was measured with a vertical stadiometer as participants stood barefoot on a flat platform. All anthropometric measurements were conducted in accordance with standardized procedures.

Blood pressure was assessed using an Omron HEM-7200 monitor following the standardized CHARLS protocol. Participants were instructed to sit, relax, and remain quiet during the measurement, with the left arm supported on a flat surface at heart level. The cuff was placed on the left upper arm, and blood pressure was measured three times at 45-second intervals. Systolic blood pressure, diastolic blood pressure, and pulse rate were recorded at each measurement, and the baseline blood pressure value was calculated as the average of the three readings.

### Assessment of CVD

The primary outcome of this study was the incidence of CVD during the follow-up period from baseline to 2018. In the CHARLS survey, participants were asked standardized questions during the interviews: “Have you been diagnosed with a stroke or a heart condition (e.g., heart attack, coronary heart disease, angina, congestive heart failure, or other heart problems) by a doctor?” and “Are you currently undergoing any treatments (e.g., Chinese traditional medicine, Western modern medicine, other treatments) for stroke, heart conditions, or their complications?” Consistent with previous studies^30,31^, participants were classified as having CVD if they self-reported receiving a doctor’s diagnosis of stroke or heart disease, or if they reported undergoing specific treatments for these conditions.

### Assessment of covariates

Information on sociodemographic characteristics (age, gender, educational level [elementary school or below; middle school; college or above], residence [urban or rural], and marital status), lifestyle factors (smoking and alcohol consumption), anthropometric measurements (BMI), clinical measurements (SBP and diastolic blood pressure [DBP]), and medical history (hypertension, diabetes, dyslipidemia, kidney disease, liver disease, as well as treatments for hypertension, diabetes, and dyslipidemia) was collected. Laboratory tests included fasting blood glucose (FBG), glycated hemoglobin A1c (HbA1c), total cholesterol (TC), triglycerides (TG), high-density lipoprotein cholesterol (HDL-c), and low-density lipoprotein cholesterol (LDL-c).

Hypertension was defined as self-reported history of hypertension, use of antihypertensive medications, systolic blood pressure ≥ 140 mmHg, or diastolic blood pressure ≥ 90mmHg^32^. Participants were classified as having diabetes if they self-reported a history of diabetes, had received any antihyperglycemic treatment, or had baseline FBG levels ≥ 126 mg/dL or HbA1c ≥ 6.5%^33^. Dyslipidemia was identified based on self-reported history of dyslipidemia, use of lipid-lowering medications, or laboratory results indicating TG ≥ 150 mg/dL, TC ≥ 240 mg/dL, HDL-c < 40 mg/dL, or LDL-c ≥ 160 mg/dL^34^. Other medical conditions were determined through self-reported history or receipt of specific treatments.

### Statistical analysis

Participants were categorized into four groups based on WHtR and SBP, using thresholds of 0.5 and 140 mmHg, respectively: Group 1 (WHtR < 0.5 and SBP < 140 mmHg), Group 2 (WHtR ≥ 0.5 and SBP < 140 mmHg), Group 3 (WHtR < 0.5 and SBP ≥ 140 mmHg), and Group 4 (WHtR ≥ 0.5 and SBP ≥ 140 mmHg). Baseline characteristics of participants were summarized as follows: Continuous variables were expressed as mean ± standard deviation (SD) for normally distributed data or as median (interquartile range, IQR) for non-normally distributed data, while categorical variables were presented as numbers (percentages). Group differences in continuous variables were analyzed using one-way analysis of variance (ANOVA) for normally distributed data or the Kruskal-Wallis test for non-normally distributed data, whereas differences in categorical variables were assessed using the Pearson χ² test. The Kolmogorov-Smirnov test was applied to evaluate the normality of continuous variables, and Levene’s test was used to assess homogeneity of variances.

To maximize the sample size despite the low proportion of missing data, we used multiple imputation by chained equations (MICE) under the assumption of missing at random. Details on the missing data counts and specific imputation methods are summarized in Supplementary Table S1.

The cumulative incidence risk of CVD events was estimated using Kaplan-Meier curves, and differences between WHtR and SBP categorical groups were compared using Log-Rank tests. To evaluate the individual and combined effects of WHtR and SBP thresholds on CVD incidence across different groups, Cox proportional hazards models were fitted, including an unadjusted crude model, a partially adjusted model, and a fully adjusted model. Hazard ratios (HRs) and their 95% confidence intervals (CIs) were reported. The adjustment details were as follows: the crude model included no covariate adjustments; Model 1 adjusted for age and gender; Model 2 further adjusted for residence, educational level, marital status, smoking status, and alcohol consumption; and Model 3 additionally included HbA1c, HDL-c, LDL-c, diabetes, and dyslipidemia to account for baseline metabolic status. These variables were entered as metabolic covariates to evaluate whether the associations of combined WHtR and SBP categories with incident CVD remained after further adjustment for concurrent metabolic abnormalities. Because some of these covariates may also overlap with pathways linking WHtR and SBP to CVD, Model 3 was interpreted as an expanded and more conservative model rather than a pure total-effect model. To formally assess interaction between WHtR and SBP, multiplicative interaction was evaluated by including a product term (WHtR × SBP) in Model 3. Additive interaction was further assessed using the relative excess risk due to interaction (RERI), attributable proportion due to interaction (AP), and synergy index (S).

Additionally, to explore potential heterogeneity in the association between combined WHtR and SBP thresholds and incident CVD, subgroup analyses were conducted. Participants were categorized into subgroups based on baseline characteristics, including age (< 60 years vs. ≥60 years), gender, smoking status, alcohol consumption status, and the presence of hypertension, diabetes, or dyslipidemia. Sensitivity analyses were performed to further assess the robustness of the findings by excluding participants with incomplete covariate data, those receiving antihypertensive or lipid-lowering treatments at baseline, and those with a follow-up duration of less than 2 years. All statistical analyses were conducted using R software (version 4.4.1). Two-sided tests were applied, and *P* < 0.05 was considered statistically significant.

## Supplementary Information

Below is the link to the electronic supplementary material.

Supplementary Material 1

## Data Availability

The data used in this study can be obtained from the publicly accessible CHARLS database (http://charls.pku.edu.cn).
