# Supplementary material for: Association of combined waist-to-height ratio and systolic blood pressure categories with incident cardiovascular disease in middle-aged and older adults
Source: Sci Rep. 2026 May 28;16:24555. doi: 10.1038/s41598-026-55572-z (PMC13454195; doi:10.1038/s41598-026-55572-z)
Supplement: Supplementary file 1 [file 41598_2026_55572_MOESM1_ESM.doc]

**Supplementary Material**

| **Variable** | **Number of missing data** | **Percentage of missing data (%)** | **Imputation methods** |
| --- | --- | --- | --- |
| Residence | 3 | 0.04 | logreg |
| BMI | 19 | 0.28 | pmm |
| Smoking | 15 | 0.22 | polyreg |
| Drinking | 4 | 0.06 | polyreg |
| Diabetes | 48 | 0.71 | logreg |
| Hypertension | 34 | 0.50 | logreg |
| Dyslipidemia | 135 | 1.99 | logreg |
| Kidney disease | 23 | 0.34 | logreg |
| Liver disease | 30 | 0.44 | logreg |
| Antihyperglycemic treatment | 49 | 0.72 | logreg |
| Antihypertensive treatment | 35 | 0.52 | logreg |
| Lipid-lowering treatment | 136 | 2.01 | logreg |
| HbA1C | 64 | 0.95 | pmm |
| FBG | 116 | 1.71 | pmm |
| TG | 108 | 1.60 | pmm |
| TC | 110 | 1.62 | pmm |
| HDL-c | 105 | 1.55 | pmm |
| LDL-c | 118 | 1.74 | pmm |

**Table S1.** The proportion of missing covariates and imputation methods. BMI, body mass index; HbA1c, glycosylated hemoglobin A1c; FBG, fasting blood glucose; TG, triglycerides; TC, total cholesterol; HDL-c, high-density lipoprotein cholesterol; LDL-C, low-density lipoprotein cholesterol.

|  | **Non- CVD** | **CVD** | **P value** |
| --- | --- | --- | --- |
| N | 5386 | 1384 |  |
| Gender, n (%) |  |  | <0.001 |
| Male | 2558 (47.5) | 581 (42.0) |  |
| Female | 2828 (52.5) | 803 (58.0) |  |
| Age, years | 57.75 ± 8.79 | 60.17 ± 8.87 | <0.001 |
| Marital status, n (%) |  |  | 0.132 |
| Married | 4600 (85.4) | 1159 (83.7) |  |
| Other | 786 (14.6) | 225 (16.3) |  |
| Education level, n (%) |  |  | 0.057 |
| Elementary school or below | 3770 (70.0) | 987 (71.3) |  |
| Middle school | 1489 (27.6) | 352 (25.4) |  |
| College or above | 127 (2.4) | 45 (3.3) |  |
| Residence, n (%) |  |  | <0.001 |
| Rural | 4640 (86.1) | 1133 (81.9) |  |
| Urban | 746 (13.9) | 251 (18.1) |  |
| Smoking status, n (%) |  |  | 0.001 |
| Never | 3257 (60.5) | 869 (62.8) |  |
| Former | 391 (7.3) | 131 (9.5) |  |
| Current | 1738 (32.3) | 384 (27.7) |  |
| Drinking status, n (%) |  |  | <0.001 |
| Never | 3105 (57.6) | 851 (61.5) |  |
| Former | 390 (7.2) | 134 (9.7) |  |
| Current | 1891 (35.1) | 399 (28.8) |  |
| Hypertension, n (%) | 997 (18.5) | 489 (35.3) | <0.001 |
| Diabetes, n (%) | 228 (4.2) | 105 (7.6) | <0.001 |
| Dyslipidemia, n (%) | 303 (5.6) | 191 (13.8) | <0.001 |
| Kidney disease, n (%) | 236 (4.4) | 90 (6.5) | 0.001 |
| Liver disease, n (%) | 141 (2.6) | 53 (3.8) | 0.020 |
| Antihypertensive treatment, n (%) | 680 (12.6) | 357 (25.8) | <0.001 |
| Antihyperglycemic treatment, n (%) | 130 ( 2.4) | 72 (5.2) | <0.001 |
| Lipid-lowering treatment, n (%) | 144 (2.7) | 88 (6.4) | <0.001 |
| SBP, mmHg | 127.18 ± 20.24 | 133.79 ± 22.53 | <0.001 |
| DBP, mmHg | 74.46 ± 11.76 | 76.95 ± 12.62 | <0.001 |
| BMI, kg/m^2^ | 22.95 (20.73, 25.37) | 23.79 (21.29, 26.21) | <0.001 |
| FBG, mg/dl | 102.06 (94.14, 112.50) | 103.14 (95.04, 114.88) | <0.001 |
| HbA1c, % | 5.10 (4.90, 5.40) | 5.20 (4.90, 5.50) | <0.001 |
| TG, mg/dl | 102.66 (72.57, 149.34) | 110.62 (80.31, 161.96) | <0.001 |
| TC, mg/dl | 189.82 (167.01, 214.47) | 194.07 (170.10, 219.98) | <0.001 |
| HDL-c, mg/dl | 51.74 ± 15.37 | 50.44 ± 15.29 | 0.005 |
| LDL-c, mg/dl | 115.50 ± 34.60 | 118.76 ± 35.73 | 0.002 |
| WHtR | 0.53 (0.49, 0.58) | 0.55 (0.51, 0.60) | <0.001 |

**Table S2.** Baseline characteristics of participants classified by CVD incidence. CVD, cardiovascular disease; SBP, systolic blood pressure; DBP, diastolic blood pressure; BMI, body mass index; FBG, fasting blood glucose; HbA1c, glycosylated hemoglobin A1c; TG, triglycerides; TC, total cholesterol; HDL-c, high-density lipoprotein cholesterol; LDL-C, low-density lipoprotein cholesterol; WHtR, waist-to-height ratio.

**Fig. S1.** Kaplan-Meier plot of heart condition by WHtR and SBP thresholds. Group 1: WHtR < 0.5 and SBP < 140 mmHg; Group 2: WHtR ≥ 0.5 and SBP < 140 mmHg; Group 3: WHtR < 0.5 and SBP ≥ 140 mmHg; Group 4: WHtR ≥ 0.5 and SBP ≥ 140 mmHg. WHtR, waist-to-height ratio; SBP, systolic blood pressure.

**Fig. S2.** Kaplan-Meier plot of stroke by WHtR and SBP thresholds. Group 1: WHtR < 0.5 and SBP < 140 mmHg; Group 2: WHtR ≥ 0.5 and SBP < 140 mmHg; Group 3: WHtR < 0.5 and SBP ≥ 140 mmHg; Group 4: WHtR ≥ 0.5 and SBP ≥ 140 mmHg. WHtR, waist-to-height ratio; SBP, systolic blood pressure.

|  | **Case, n (%)** | **Crude** | **Model 1** | | **Model 2** | | **Model 3** | | |
| --- | --- | --- | --- | --- | --- | --- | --- | --- | --- |
|  |  | **HR (95% CI)** | **P value** | **HR (95% CI)** | **P value** | **HR (95% CI)** | **P value** | **HR (95% CI)** | **P value** |
| **WHtR** |  |  |  |  |  |  |  |  |  |
| WHtR<0.5 | 240 (12.0) | Reference |  | Reference |  | Reference |  | Reference |  |
| WHtR≥0.5 | 800 (16.8) | 1.438 (1.244, 1.661) | <0.001 | 1.308 (1.127- 1.517) | <0.001 | 1.255 (1.081- 1.457) | <0.001 | 1.183 (1.014- 1.381) | <0.001 |
| **SBP** |  |  |  |  |  |  |  |  |  |
| SBP<140 | 711 (14.1) | Reference |  | Reference |  | Reference |  | Reference |  |
| SBP≥140 | 329 (19.1) | 1.475 (1.294- 1.681) | <0.001 | 1.308 (1.143- 1.497) | <0.001 | 1.323 (1.156- 1.515) | <0.001 | 1.263 (1.103- 1.447) | <0.001 |
| **WHtR and SBP** |  |  |  |  |  |  |  |  |  |
| Group 1 | 187 (11.2) | Reference |  | Reference |  | Reference |  | Reference |  |
| Group 2 | 524 (15.5) | 1.419 (1.201- 1.677) | <0.001 | 1.306 (1.101- 1.549) | 0.002 | 1.251 (1.054- 1.484) | 0.011 | 1.201 (1.008- 1.430) | 0.041 |
| Group 3 | 53 (16.1) | 1.595 (1.176- 2.164) | 0.003 | 1.413 (1.039- 1.921) | 0.028 | 1.449 (1.065- 1.972) | 0.018 | 1.453 (1.067- 1.977) | 0.018 |
| Group 4 | 276 (19.8) | 1.953 (1.622- 2.352) | <0.001 | 1.621 (1.339- 1.964) | <0.001 | 1.574 (1.300- 1.907) | <0.001 | 1.444 (1.185- 1.759) | <0.001 |

**Table S3.** Association of WHtR and SBP with the risk of heart condition. Group 1: WHtR < 0.5 and SBP < 140 mmHg; Group 2: WHtR ≥ 0.5 and SBP < 140 mmHg; Group 3: WHtR < 0.5 and SBP ≥ 140 mmHg; Group 4: WHtR ≥ 0.5 and SBP ≥ 140 mmHg. WHtR, waist-to-height ratio; SBP, systolic blood pressure; HR, hazard ratio; CI, confidence interval. Crude: unadjusted model; Model 1: adjusted for age, gender; Model 2: adjusted for marital status, education level, residence, smoking status and drinking status based on Model 1; Model 3: additionally adjusted for HbA1c, HDL-c, LDL-c, diabetes, and dyslipidemia to account for baseline metabolic status, based on Model 2.

|  | **Case, n (%)** | **Crude** | | **Model 1** | | **Model 2** | | **Model 3** | |
| --- | --- | --- | --- | --- | --- | --- | --- | --- | --- |
|  |  | **HR (95% CI)** | **P value** | **HR (95% CI)** | **P value** | **HR (95% CI)** | **P value** | **HR (95% CI)** | **P value** |
| **WHtR** |  |  |  |  |  |  |  |  |  |
| WHtR<0.5 | 85 (4.3) | Reference |  | Reference |  | Reference |  | Reference |  |
| WHtR≥0.5 | 383 (8.0) | 1.957 (1.547, 2.475) | <0.001 | 2.116 (1.664- 2.690) | <0.001 | 2.084 (1.636- 2.654) | <0.001 | 1.788 (1.393- 2.294) | <0.001 |
| **SBP** |  |  |  |  |  |  |  |  |  |
| SBP<140 | 269 (5.3) | Reference |  | Reference |  | Reference |  | Reference |  |
| SBP≥140 | 199 (11.5) | 2.433 (2.025- 2.922) | <0.001 | 2.141 (1.772- 2.586) | <0.001 | 2.129 (1.762- 2.573) | <0.001 | 1.950 (1.611- 2.359) | <0.001 |
| **WHtR and SBP** |  |  |  |  |  |  |  |  |  |
| Group 1 | 61 (3.66) | Reference |  | Reference |  | Reference |  | Reference |  |
| Group 2 | 208 (6.16) | 1.732 (1.302- 2.304) | <0.001 | 1.909 (1.429- 2.550) | <0.001 | 1.866 (1.394- 2.497) | <0.001 | 1.658 (1.233- 2.230) | <0.001 |
| Group 3 | 24 (7.27) | 2.284 (1.424- 3.663) | <0.001 | 1.971 (1.226- 3.168) | 0.005 | 1.947 (1.210- 3.135) | 0.006 | 1.935 (1.202- 3.114) | 0.007 |
| Group 4 | 175 (12.5) | 3.928 (2.935- 5.257) | <0.001 | 3.772 (2.799- 5.084) | <0.001 | 3.670 (2.721- 4.950) | <0.001 | 3.041 (2.236- 4.137) | <0.001 |

**Table S4.** Association of WHtR and SBP with the risk of stroke. Group 1: WHtR < 0.5 and SBP < 140 mmHg; Group 2: WHtR ≥ 0.5 and SBP < 140 mmHg; Group 3: WHtR < 0.5 and SBP ≥ 140 mmHg; Group 4: WHtR ≥ 0.5 and SBP ≥ 140 mmHg. WHtR, waist-to-height ratio; SBP, systolic blood pressure; HR, hazard ratio; CI, confidence interval. Crude: unadjusted model; Model 1: adjusted for age, gender; Model 2: adjusted for marital status, education level, residence, smoking status and drinking status based on Model 1; Model 3: additionally adjusted for HbA1c, HDL-c, LDL-c, diabetes, and dyslipidemia to account for baseline metabolic status, based on Model 2.

|  | **Case, n (%)** | **Crude** | | **Model 1** | | **Model 2** | | **Model 3** | |
| --- | --- | --- | --- | --- | --- | --- | --- | --- | --- |
|  |  | **HR (95% CI)** | **P value** | **HR (95% CI)** | **P value** | **HR (95% CI)** | **P value** | **HR (95% CI)** | **P value** |
| Group 1 | 225 (14.4) | Reference |  | Reference |  | Reference |  | Reference |  |
| Group 2 | 656 (20.4) | 1.455 (1.251- 1.693) | <0.001 | 1.402 (1.201- 1.637) | <0.001 | 1.351 (1.156- 1.579) | <0.001 | 1.265 (1.079- 1.483) | 0.004 |
| Group 3 | 71 (23.1) | 1.857 (1.421- 2.426) | <0.001 | 1.631 (1.243- 2.141) | <0.001 | 1.642 (1.247- 2.161) | <0.001 | 1.648 (1.253- 2.168) | <0.001 |
| Group 4 | 370 (28.3) | 2.211 (1.873, 2.609) | <0.001 | 1.905 (1.600- 2.268) | <0.001 | 1.852 (1.555- 2.206) | <0.001 | 1.647 (1.375- 1.972) | <0.001 |

**Table S5.** Association of WHtR and SBP with the risk of CVD incidence after excluding participants with any missing covariates data before multiple imputation. Group 1: WHtR < 0.5 and SBP < 140 mmHg; Group 2: WHtR ≥ 0.5 and SBP < 140 mmHg; Group 3: WHtR < 0.5 and SBP ≥ 140 mmHg; Group 4: WHtR ≥ 0.5 and SBP ≥ 140 mmHg. WHtR, waist-to-height ratio; SBP, systolic blood pressure; CVD, cardiovascular disease; HR, hazard ratio; CI, confidence interval. Crude: unadjusted model; Model 1: adjusted for age, gender; Model 2: adjusted for marital status, education level, residence, smoking status and drinking status based on Model 1; Model 3: additionally adjusted for HbA1c, HDL-c, LDL-c, diabetes, and dyslipidemia to account for baseline metabolic status, based on Model 2.

|  | **Case, n (%)** | **Crude** | | **Model 1** | | **Model 2** | | **Model 3** | |
| --- | --- | --- | --- | --- | --- | --- | --- | --- | --- |
|  |  | **HR (95% CI)** | **P value** | **HR (95% CI)** | **P value** | **HR (95% CI)** | **P value** | **HR (95% CI)** | **P value** |
| Group 1 | 225 (13.7) | Reference |  | Reference |  | Reference |  | Reference |  |
| Group 2 | 657 (19.8) | 1.490 (1.280- 1.733) | <0.001 | 1.444 (1.237- 1.685) | <0.001 | 1.392 (1.192- 1.626) | <0.001 | 1.313 (1.121- 1.539) | <0.001 |
| Group 3 | 71 (22.5) | 1.815 (1.390- 2.370) | <0.001 | 1.600 (1.223- 2.094) | <0.001 | 1.622 (1.239- 2.124) | <0.001 | 1.624 (1.240- 2.127) | <0.001 |
| Group 4 | 366 (27.3) | 2.214 (1.875, 2.614) | <0.001 | 1.920 (1.618- 2.279) | <0.001 | 1.866 (1.573- 2.215) | <0.001 | 1.670 (1.400- 1.993) | <0.001 |

**Table S6.** Association of WHtR and SBP with the risk of CVD incidence after excluding participants with follow duration less than two years. Group 1: WHtR < 0.5 and SBP < 140 mmHg; Group 2: WHtR ≥ 0.5 and SBP < 140 mmHg; Group 3: WHtR < 0.5 and SBP ≥ 140 mmHg; Group 4: WHtR ≥ 0.5 and SBP ≥ 140 mmHg. WHtR, waist-to-height ratio; SBP, systolic blood pressure; CVD, cardiovascular disease; HR, hazard ratio; CI, confidence interval. Crude: unadjusted model; Model 1: adjusted for age, gender; Model 2: adjusted for marital status, education level, residence, smoking status and drinking status based on Model 1; Model 3: additionally adjusted for HbA1c, HDL-c, LDL-c, diabetes, and dyslipidemia to account for baseline metabolic status, based on Model 2.

|  | **Case, n (%)** | **Crude** | | **Model 1** | | **Model 2** | | **Model 3** | |
| --- | --- | --- | --- | --- | --- | --- | --- | --- | --- |
|  |  | **HR (95% CI)** | **P value** | **HR (95% CI)** | **P value** | **HR (95% CI)** | **P value** | **HR (95% CI)** | **P value** |
| Group 1 | 219 (13.8) | Reference |  | Reference |  | Reference |  | Reference |  |
| Group 2 | 527 (18.1) | 1.342 (1.147- 1.572) | <0.001 | 1.306 (1.111- 1.535) | 0.001 | 1.276 (1.085- 1.501) | 0.003 | 1.226 (1.038- 1.447) | 0.016 |
| Group 3 | 57 (21.5) | 1.733 (1.295- 2.319) | <0.001 | 1.500 (1.118- 2.013) | 0.007 | 1.528 (1.137- 2.052) | 0.005 | 1.535 (1.143- 2.061) | 0.004 |
| Group 4 | 194 (22.4) | 1.795 (1.480, 2.178) | <0.001 | 1.542 (1.264- 1.882) | <0.001 | 1.520 (1.246- 1.856) | <0.001 | 1.440 (1.176- 1.764) | <0.001 |

**Table S7.** Association of WHtR and SBP with the risk of CVD incidence after excluding participants receiving antihypertensive treatment or lipid-lowering treatment. Group 1: WHtR < 0.5 and SBP < 140 mmHg; Group 2: WHtR ≥ 0.5 and SBP < 140 mmHg; Group 3: WHtR < 0.5 and SBP ≥ 140 mmHg; Group 4: WHtR ≥ 0.5 and SBP ≥ 140 mmHg. WHtR, waist-to-height ratio; SBP, systolic blood pressure; CVD, cardiovascular disease; HR, hazard ratio; CI, confidence interval. Crude: unadjusted model; Model 1: adjusted for age, gender; Model 2: adjusted for marital status, education level, residence, smoking status and drinking status based on Model 1; Model 3: additionally adjusted for HbA1c, HDL-c, LDL-c, diabetes, and dyslipidemia to account for baseline metabolic status, based on Model 2.
